# Retraction: Metformin protects from rotenone–induced nigrostriatal neuronal death in adult mice by activating AMPK-FOXO3 signaling and mitigation of angiogenesis

**DOI:** 10.3389/fnmol.2026.1781967

**Published:** 2026-01-13

**Authors:** Frontiers Editorial Office

The journal retracts the 18 June 2020 article cited above.

Following publication, concerns were raised regarding the integrity of the images in the published figures. Image duplication concerns were identified in Figure 7A.

The authors failed to provide a satisfactory explanation during the investigation, which was conducted in accordance with Frontiers' policies. As a result, the data and conclusions of the article have been deemed unreliable, and the article has been retracted.

This retraction was approved by the Chief Editors of Frontiers in Molecular Neuroscience and the Chief Executive Editor of Frontiers. The authors do not agree to this retraction.

Frontiers would like to thank the concerned readers who contacted us regarding the published article.

